# A new set of highly efficient primers for COI amplification in rotifers

**DOI:** 10.1080/23802359.2021.1878951

**Published:** 2021-02-19

**Authors:** Yanan Zhang, Shaolin Xu, Chenghe Sun, Henri Dumont, Bo-Ping Han

**Affiliations:** Department of Ecology and Institute of Hydrobiology, Jinan University, Guangzhou, China

**Keywords:** COI, primer, rotifera, DNA barcode, phylogeny

## Abstract

Rotifers are a small-sized but key group of freshwater zooplankters with high species richness, linking primary producers to higher consumers in aquatic food webs. DNA barcoding has been widely used in exploring its biodiversity, cryptic speciation and phylogeny. However, the inefficiency of universal primers to amplify COI of rotifers hinders our understanding of their species richness and genetic diversity. Here, we develop a new pair of primers, 30 F and 885 R, to amplify the COI gene of rotifers. We used 22 species to test their PCR success rate and found that the new pair of primers was more efficient (86%) than two pairs of universal primers, namely, dgLCO and dgHCO (32%), and Folmer primers (59%). The new primers will allow the barcoding of groups that were so far difficult to sequence and will contribute to clarify species diversity and phylogeny of rotifers.

## 1. Introduction

Rotifers are key invertebrates in freshwater ecosystems and play an important role in aquatic food webs by transferring matter and energy from producers to higher consumers (Rublee 1998). They are highly sensitive to changes in environments and become useful indicators of water quality (Duggan et al. 2001). It is of great significance to correctly document their species composition, essential in biodiversity monitoring, and to monitor ecological change in aquatic ecosystems (Sladecek 1983). Due to their short generation time and facultative cyclic parthenogenesis, some species are used as model organisms in ecology, toxicology and evolutionary biology (Radix et al. 2002; Campillo et al. 2011; Flot et al. 2013). Species identification is a condition to evaluate biodiversity and investigate mechanisms that maintain species diversity (Proios et al. 2014). However, many rotifer species show strong morphological variability while, and conversely, some complex cryptic species are similar in morphology (Derry et al. 2003; Segers 2007). Thus, the identification of some rotifers species only based on morphology is controversial, and studies of rotifer ecology and species distribution face the problem of inaccurate species identification (Alcántara-Rodríguez et al. 2012; Garcia-Morales and Elias-Gutierrez 2013; Elias-Gutierrez et al. 2019)

After Kumazaki et al. (1982) reported the first 5S rRNA sequence for rotifers, a large number of DNA such sequences have been published. DNA sequencing has been widely used in the ecological and phylogeny of rotifers. Among the markers used, mitochondrial cytochrome oxidase subunit I (COI) is one of the most popular, it has a high mutation rate, relatively reliable primer binding region, and high copy number per cell (Hebert et al. 2003; Birky 2007). COI barcoding has become popular in the identification of microfauna such as rotifers (Garcia-Morales and Elias-Gutierrez 2013). Mills et al. (2017) collected the sequences of COI and internal transcribed spacer-1 (ITS1) of *Brachionus plicatilis* O.F. Müller, 1786 and used DNA taxonomic methods to claim 15 cryptic species within the complex, including *Brachionus asplanchnoidis* Charin, 1947, *Brachionus manjavacas* Fontaneto, Giordani, Melone & Serra, 2007, *B. plicatilis* sensu stricto (*s.s.*) and *Brachionus rotundiformis* Tschugunoff, 1921. Gomez et al (2002) used the ITS1 and COI sequences of rotifer resting eggs from 27 salt lakes plus lakes from four continents to find that the *B. plicatilis* complex began diversifying many millions of years ago and showed relatively high levels of morphological stasis. HCO1490/LCO2198 primers (‘Folmer’ primers) are the most common universal primers that amplify common rotifers groups such as *Brachionus* and *Keratella*, Still, these primers often fail or poorly amplify, compromising practical application (Folmer et al. 1994; Sørensen and Giribet 2006; Geller et al. 2013; Moreno et al. 2017). Meyer et al (2005) obtained dgLCO/dgHCO primers by modifying the ‘Folmer’ primer, currently used to amplify the COI sequence of the *B. plicatilis* complex (Rico-Martínez et al. 2013). However, the dgLCO/dgHCO primers did not largely improve the amplifying efficiency. New sets of universal and highly efficient primers for the COI amplification specifically for rotifers remain in development (Birky 2007). For example, Wilts et al. (2012) using the primers COI-F/COI-R investigated the phylogeny of *Proales daphnicola* Thompson, 1892 (Proalidae) and relocated it into *Epiphanes*. To avoid the problems of COI amplification, Hirai (2013) proposed to use 28S rRNA instead. However, the mutation rate of 28S rRNA is much slower than that of COI and does not provide sufficient resolution to ascertain the genetic relationships between related species (Elias-Gutierrez et al. 2018).

In the present study, we filtered mitochondrial sequences of rotifers from a metagenomic data set and then assembled them. Using nine incomplete mitochondrial genome sequences from the genera *Brachionus* and *Keratella*, a new pair of primers 30 F/885R was designed to amplify the COI barcode fragment. The primers improved the success rate of COI amplification in rotifers, greatly help the recovery of barcodes from difficult groups and facilitate work on diversity and phylogeny in rotifers.

## 2. Materials and methods

### 2.1. Sample collection and DNA extraction

Specimens were sampled from the following eight reservoirs located in Guangzhou (southern China) in September 2019: Nanhu, Longdong, Dashan, Shuisheng, Yujiazhuang, Shanjiao, Zengtang and Muqiang. The reservoirs were selected to cover diverse habitats and species diversity at a larger regional scale. Rotifers were collected using a 38 μm plankton net from surface waters, then fixed with BBI’s DNA-EZ Reagents F DNA tissue sample storage solution and stored in a refrigerator at 4 °C. All collected specimens and extracts were stored at −20 °C at the Institute of Hydrobiology, Jinan University, Guangzhou, China.

Specimens were identified morphologically under a stereomicroscope. In total, 22 species including common dominant and rare species were identified: *Keratella cochlearis* Gosse, 1851, *Keratella tropica* Apstein, 1907, *Keratella tecta* Gosse, 1851, *Brachionus diversicornis* Daday, 1883, *Brachionus falcatus* Zacharias, 1898, *Brachionus caudatus* Barrois-Daday, 1894, *Brachionus urceolaris* O.F. Müller, 1773, *Brachionus angularis* Gosse, 1851, *Brachionus quadridentatus* Herman, 1783, *Brachionus forficula* Wierzejski, 1891, *Asplanchna priodonta* Gosse, 1850, *Asplanchna brightwelli* Gosse, 1850, *Polyarthra vulgaris* Carlin, 1943, *Trichocerca dixonnuttalli* Jennings, 1903, *Trichocerca capucina* Wierzejski & Zacharias, 1893, *Trichocerca cylindrica* Imhof, 1891, *Synchaeta oblonga* Ehrenberg, 1832, *Lecane bulla* Gosse, 1851, *Lecane* sp1., *Lecane* sp2., *Ascomorpha ovalis* Bergendal, 1892 and *Ploesoma hudsoni* Imhof, 1891. Prior to NDA extraction for each species, all individuals were washed with MilliQ water, and then single individuals were kept in a 0.2 mL tube. 3 μl Proteinase K and 30 μl Chelex resin (BioRad, Hemel Hempstead, UK) was added into the tube for DNA extraction. The tube was centrifuged at 8000 rpm for 1 min and finally put into the PCR instrument. The DNA samples were incubated at 56 °C for 60 min, at 99 °C for 10 min and stored at 12 °C. All samples were stored at 4 °C and used directly in PCR reactions, or at −20 °C for long storage. Finally, the samples were centrifuged at 10,000 rpm for 2 min and the supernatant was directly used in each PCR reaction. These DNAs were used for primer comparison.

### 2.2. Metagenomic sequencing and data analysis

Rotifers from eight reservoirs were mixed and their metagenomic DNA was extracted with Megen's HiPure Tissue DNA Mini Kit after a standard operating procedure. The DNA samples were sent to BioMarker Company for sequencing with HiSeq PE300 sequester (Ilumina Inc., San Di-Ego, CA, USA). A set of 40 G data from sequencing was filtered to remove the connector and low-quality sequences, Megahit default parameters were used to assemble the metagenome.

### 2.3. Primer design

The assembled sequences with a length of over 500 bp were retained and mitochondrial sequences were screened by Diamond (Putz and Brandenburg 2006). Diamond reference library was constructed from the protein sequences of rotifer mitochondrial cox1 genes from NCBI. From the assembled sequences in Megahit (Li et al. 2015), we screened nine partial mitochondrial genome sequences in the genera *Brachionus* and *Keratella*. In Geneious 10.2 (https://www.geneious.com), multiple sequence alignment was performed on the above nine sequences and degenerate primers were designed: 30 F and 885 R (Table 1). The mafft algorithm is used for multiple sequence alignment, and the primers were designed with the default parameters in Geneious 10.2. In silico validation was run on the new primer to species already sequenced and available in GenBank. All COI sequences of rotifers in Genbank were downloaded and clustered with cd-hit with a similarity threshold set to 0.9. The clustered file was then aligned with mafft, and our new primer was then mapped to the aligned file manually.

**Table 1.** Sequences information of three primers used in this study.

Primer	Nucleotide sequence (5’-3’)	GC (%)	*T*m (°C)	Size (bp)
30F	F:HACTAATCAYAARGATATTGGWAC	25.0	51	850
885R	R:RAACATATGATGAGCYCAWACAAT	29.2		
LCO2198	F:GGTCAACAAATCATAAAGATATTGG	32.0	51	700
HCO1490	R:TAAACTTCAGGGTGACCAAAAAATCA	34.6		
dgLCO	F:GGTCAACAAATCATAAAGAYATYGG	32.0	47	700
dgHCO	R:TAAACTTCAGGGTGACCAAARAAYCA	34.6		

### 2.4. PCR and sequencing

We used the extracted DNA of each species as template to amplification to compare the amplification success rate of three sets of primers. The total amplification volume of primer 30 F/885R was 30 μl, including 15 μl 2 × HieffTM PCR Master Mix (With Dye), 11 μl ddH_2_O, 0.5 μl of 100 μM solution of each primer and 3 μl DNA, respectively. The amplification conditions were: initial denaturation 2 min at 98

°C, followed by six cycles of [ 95 °C for 30 s, 54 °C for 40 s (−0.5 °C/each cycle), 72 °C for 30 s], and then 36 cycles of [95 °C for 30 s, 51 °C for 40 s, 72 °C for 30 s] and final extension of 72 °C for 2 min. The amplification systems and conditions of HCO1490 and LCO2198 refer to Folmer et al. (1994), the amplification systems and conditions of dgLCO and dgHCO refer to Meyer et al. (2005). After electrophoresis, visually positive PCR products were selected for purification and double-terminal sequencing.

### 2.5. Data analysis and validation

To obtain consistent forward and reverse sequences, the sequencing results of positive PCR were assembled in Geneious 10.2. We used BLAST in NCBI database to preliminarily verify the reliability of the sequences. PCR reaction was considered successful only when BLAST results indicated that the consistent sequence was from rotifers. Finally, the amplification success rates of three pairs of primers for all the tested rotifers were calculated and compared.

## 3. Results

We sequenced 22 rotifers species, of which 20 were identified at the species level and two only to the genus level. The rotifers from the eight reservoirs belong to six families and nine genera. There are seven species of *Brachionus*, three of *Keratella*, three of *Lecane*, three of *Trichocerca*, two of *Asplanchna*, and one of *Polyarthra*, *Synchaeta*, *Ascomorpha* and *Ploesoma*. Three pairs of primers, 30 F/885R, LCO2198/HCO1490 and dgLCO/dgHCO, were used to amplify COI sequences of each species. All sequences obtained were uploaded to the GenBank database (accession number:MT908928-MT908946). Nine partial mitochondrial genome sequences of *Branchionus* and *Keratella* and the Diamond reference library were deposited on Github (https://github.com/ShaolinXU/New-COX1-primers-for-rotifera).

**Figure 1.**
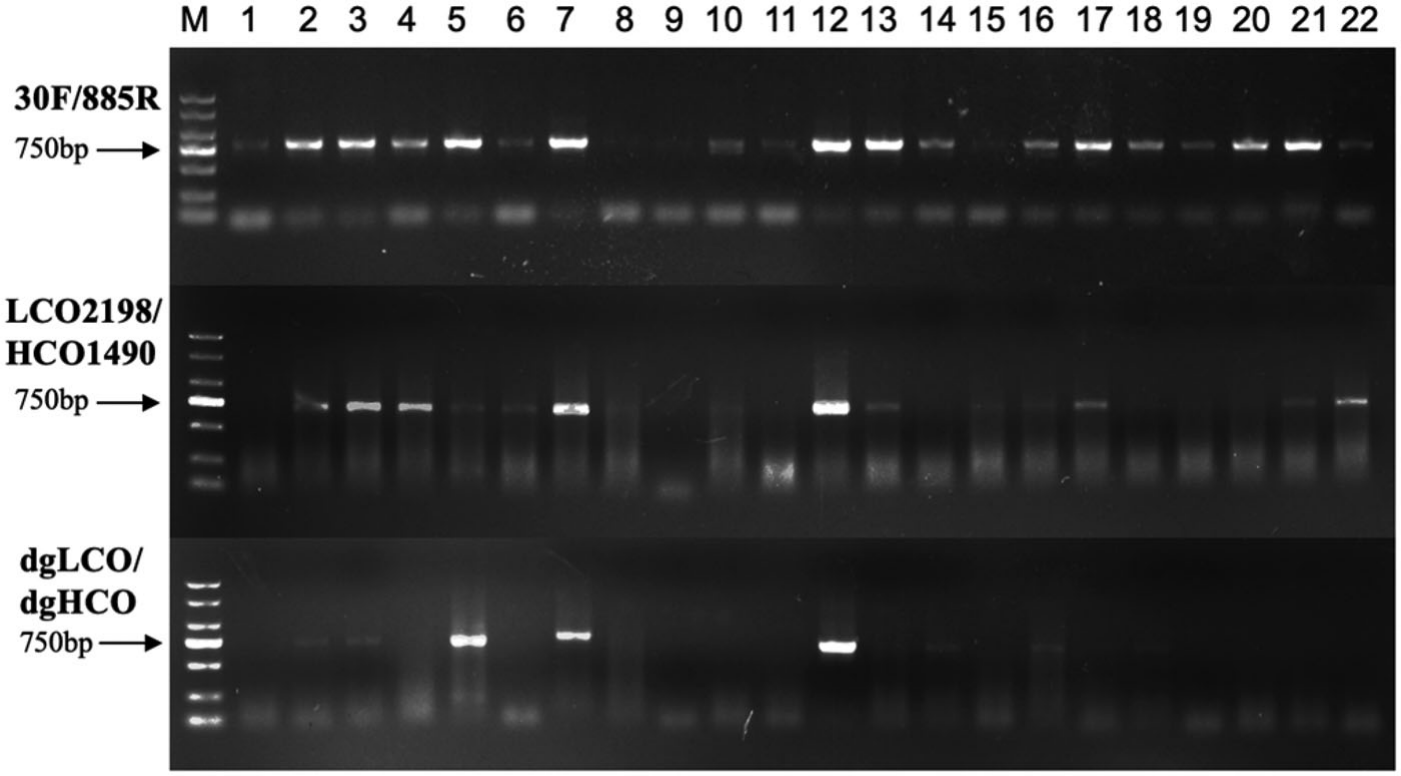
Electrophoretogram of PCR amplification for 22 rotifers: (1) *K. cochlearis*, (2) *K. tropica*, (3) *K. tecta*, (4) *B. diversicornis*, (5) *B. falcatus*, (6) *B. caudatus*, (7) *B. urceolaris*, (8) *B. angularis*, (9) *B. quadridentatus*, (10) *B. forficula*, (11) *A. priodonta*, (12) *A. brightwelli*, (13) *P. vulgaris*, (14) *T. dixonnuttalli*, (15) *T. capucina*, (16) *T. cylindrica*, (17) *S. oblonga*, (18) *L. bulla*, (19) *Lecane* sp1., (20) *Lecane* sp2., (21) *A. ovalis*, (22) *P. hudsoni*.

**Figure 2.**
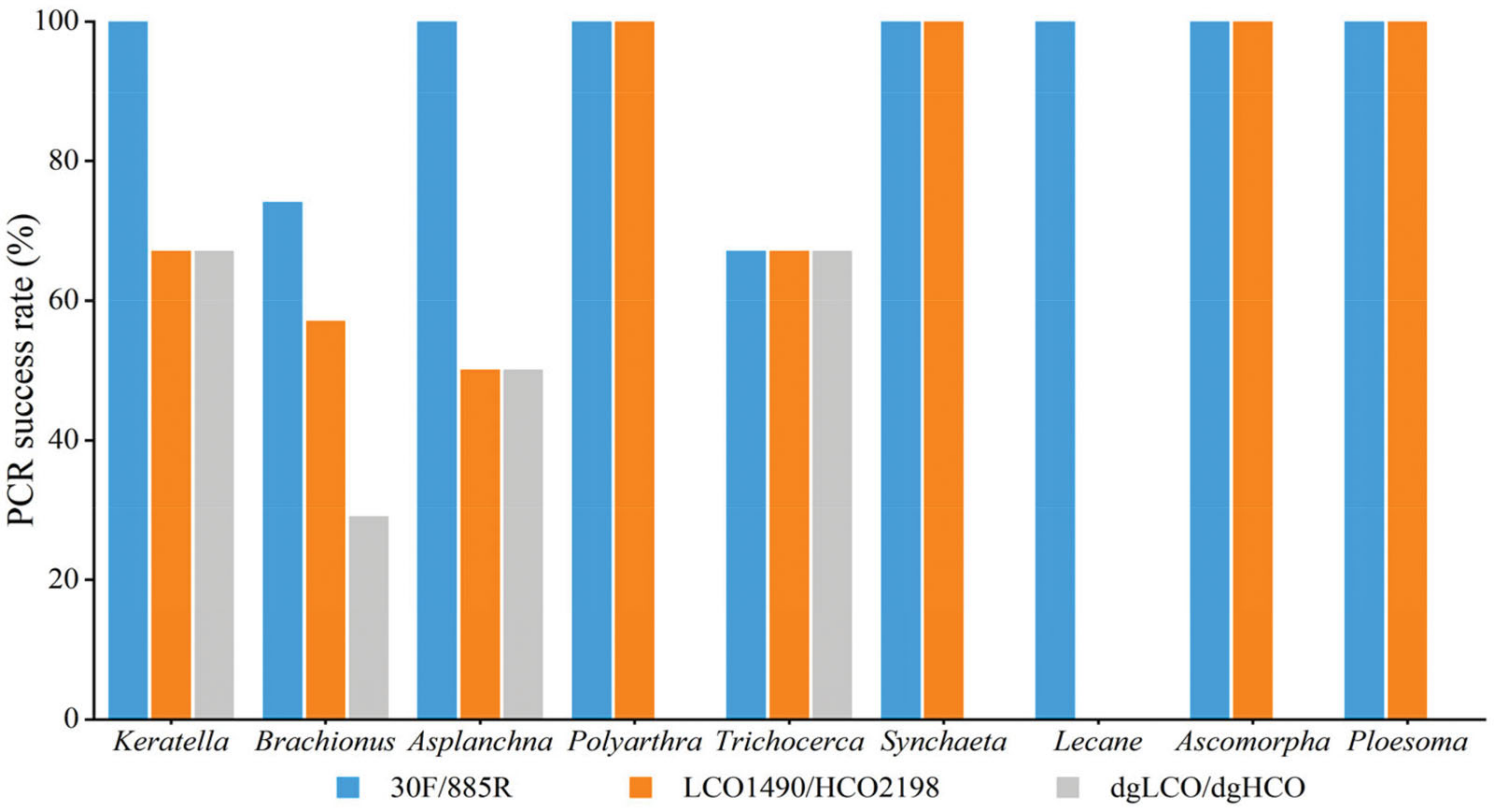
PCR success rates of three primers in nine genera rotifers (*Keratella*, *Brachionus*, *Asplanchna*, *Polyarthra*, *Trichocera*, *Synchaeta*, *Lecane*, *Ascomorpha*, *Ploesoma*).

The new pair of primers amplified target fragment successfully with clear bands (Figure 1), and it was superior to LCO2198/HCO1490 and dgLCO/dgHCO in amplification specificity. 30 F/885R had higher PCR success rate (86%) than LCO2198/HCO1490 (59%) and dgLCO/dgHCO (32%) (Figure 2). At the genus level, 30 F/885R amplified the COI fragment of *Lecane* which LCO2198/HCO1490 failed and that of *Lecane*, *Polyarthra*, *Synchaeta* and *Ploesoma* which dgLCO/dgHCO failed.

**Figure 3.**
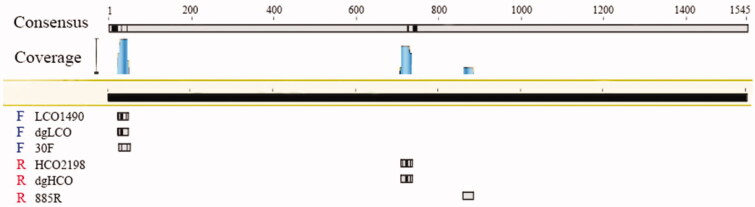
The positions of primers of different rotifers on the COI.

Moreover, we compared the positions of these three pair of primers in a complete COI sequence (Figure 3). The difference in amplification efficiency of these primers mainly depends on the base sequences and binding position. First, the degeneracy of 30 F and 885 R is twenty-four and eight, respectively, while the degeneracy of dgLCO and dgHCO are four, and the degeneracy of LCO2198 and HCO1490 are zero (Table 1). In the forward primer 30 F, there is a degenerate base ‘H’ (‘A’ or ‘C’ or ‘T’), while the corresponding base of forward primer LCO2198 and forward dgLCO is ‘A’. Second, the binding regions of the forward primers and templates of these three pairs of primers are in the range of 20–50 bp of the reference sequence (Figure 3). However, reverse primers bind to different position. The binding region of the other primers is 700–750 bp except that 885 R binding region is between 860 bp and 880 bp.

The result of in silico validation was in concordance with a comparison of nucleotide composition among the three tested primers, most variable site at the binding region are paired with degenerated sites within 30 F/885R (Figures S1 and S2).

## 4. Discussion

Rotifers distribute in a wide range of continental water bodies, from hypersaline to freshwater, and have high species richness in most freshwaters. With a high mutation rate, high phylogenetic resolution and abundant reference sequence, COI sequence has become the most widely used genetic maker in this group (Knowlton and Weigt 1998; Wares and Cunningham 2001; Ratnasingham and Hebert 2007; Geller et al. 2013). However, the lower success rate of the existing universal primers limits us to amply COI and understand the species and genetic diversity of the rotifers. The published COI sequences mainly come from *Brachionus* and *Lecane* in Europe and America (Garcia-Morales and Elias-Gutierrez 2013). High amplification efficiency of DNA barcoding not only allows us to document deeply species and genetic diversity of rotifers but also extends our investigation in less studied regions.

Although LCO2198/HCO1490 and dgLCO/dgHCO were all able to amplify COI from *Brachionus*, *Keratella*, *Asplanchna* and *Trichocerca*, the 30 F/885R had much higher success rate. The COI of some species like *B. forficula* and *K. cochlearis* can only be amplified by the new pair of primers. High degeneracy and unique binding region of the reverse primer are the two key factors responding for the difference in efficiency among the three tested primer pairs. By applying a method of touchdown PCR, we decreased nonspecific amplification caused by high degeneracy in the process of PCR. In our in-silico validation, several sites at 5-end of 30 F are mismatched with sequence of some rotifer species, but high match rates at the 3-end of 30 F and 885 R could compensate those mismatches (Figures S1 and S2), as very few mismatches at 5-end are usually not lethal to the success of PCR. In conclusion, the new primers 30 F/885R improve the success of rotifer COI amplification and can facilitate the application of DNA barcoding in investigating rotifer diversity.

## Data Availability

Newly sequenced COI sequence in this study is openly available in NCBI with GenBank number: MT908928 to MT908946. Diamond reference library, 9 partial mitochondrial genomes of rotifera and supplementary information can be found at https://github.com/ShaolinXU/New-COX1-primers-for-rotifera.

## References

[CIT0001] Alcántara-Rodriguez JA, Ciros-Perez J, Ortega-Mayagoitia E, Serrania-Soto CR, Piedra-Ibarra E. 2012. Local adaptation in populations of a *Brachionus* group *plicatilis* cryptic species inhabiting three deep crater lakes in Central Mexico. Freshwater Biol. 57(4):728–740.

[CIT0002] Birky CW. 2007. Workshop on barcoded DNA: application to rotifer phylogeny, evolution, and systematics. Hydrobiologia. 593(1):175–183.

[CIT0003] Campillo S, Garcia-Roger EM, Carmona MJ, Serra M. 2011. Local adaptation in rotifer populations. Evol Ecol. 25(4):933–947.

[CIT0004] Derry AM, Hebert PDN, Prepas EE. 2003. Evolution of rotifers in saline and subsaline lakes: a molecular phylogenetic approach. Limnol Oceanogr. 48(2):675–685.

[CIT0005] Duggan IC, Green JD, Shiel RJ. 2001. Distribution of rotifers in North Island, New Zealand, and their potential use as bioindicators of lake trophic state. Hydrobiologia. 446/447:155–164.

[CIT0006] Elias-Gutierrez M, Juracka PJ, Montoliu-Elena L, Miracle MR, Petrusek A, Korinek V. 2019. Who is *Moina micrura*? Redescription of one of the most confusing cladocerans from terra typica, based on integrative taxonomy. Limnetica. 38(1):227–252.

[CIT0007] Elias-Gutierrez M, Valdez-Moreno M, Topan J, Young MR, Cohuo-Colli JA. 2018. Improved protocols to accelerate the assembly of DNA barcode reference libraries for freshwater zooplankton. Ecol Evol. 8(5):3002–3018. 29531713 10.1002/ece3.3742 PMC5838060

[CIT0008] Flot J-F, Hespeels B, Li X, Noel B, Arkhipova I, Danchin EGJ, Hejnol A, Henrissat B, Koszul R, Aury J-M, et al. 2013. Genomic evidence for ameiotic evolution in the bdelloid rotifer *Adineta vaga*. Nature. 500(7463):453–457. 23873043 10.1038/nature12326

[CIT0009] Folmer O, Black M, Hoeh W, Lutz R, Vrijenhoek R. 1994. DNA primers for amplification of mitochondrial cytochrome c oxidase subunit I from diverse metazoan invertebrates. Mol Mar Biol Biotechnol. 3(5):294–299. 7881515

[CIT0010] Garcia-Morales AE, Elias-Gutierrez M. 2013. DNA barcoding of freshwater Rotifera in Mexico: Evidence of cryptic speciation in common rotifers. Mol Ecol Resour. 13(6):n/a–1107. 10.1111/1755-0998.12080 23433240

[CIT0011] Geller J, Meyer C, Parker M, Hawk H. 2013. Redesign of PCR primers for mitochondrial cytochrome c oxidase subunit I for marine invertebrates and application in all-taxa biotic surveys. Mol Ecol Resour. 13(5):851–861. 23848937 10.1111/1755-0998.12138

[CIT0012] Gomez A, Serra M, Carvalho GR, Lunt DH. 2002. Speciation in ancient cryptic species complexes: evidence from the molecular phylogeny of *Brachionus plicatilis* (Rotifera). Evolution. 56(7):1431–1444. 12206243 10.1111/j.0014-3820.2002.tb01455.x

[CIT0013] Hebert PDN, Ratnasingham S, de Waard JR. 2003. Barcoding animal life: cytochrome c oxidase subunit 1 divergences among closely related species. Proc Roy Soc Lond B. 270:96–99. 10.1098/rsbl.2003.0025 PMC1698023 12952648

[CIT0014] Hirai J, Shimode S, Tsuda A. 2013. Evaluation of ITS2-28S as a molecular marker for identification of calanoid copepods in the subtropical western North Pacific. J Plankton Res. 35(3):644–656.

[CIT0015] Knowlton N, Weigt LA. 1998. New dates and new rates for divergence across the Isthmus of Panama. Proc R Soc Lond B. 265(1412):2257–2263.

[CIT0016] Kumazaki T, Hori H, Osawa S, Ishii N, Suzuki K. 1982. The nucleotide sequences of 5s rRNAs from a rotifer, *Brachionus plicatilis*, and two nematodes, *Rhabditis tokai* and *Caenorhabditis elegans*. Nucleic Acids Res. 10(21):7001–7004. 6891053 10.1093/nar/10.21.7001 PMC326980

[CIT0017] Li D, Liu C-M, Luo R, Sadakane K, Lam T-W. 2015. MEGAHIT: an ultra-fast single-node solution for large and complex metagenomics assembly via succinct de Bruijn graph. Bioinformatics. 31(10):1674–1676. 25609793 10.1093/bioinformatics/btv033

[CIT0019] Meyer CP, Geller JB, Paulay G. 2005. Fine scale endemism on coral reefs: archipelagic differentiation in turbinid gastropods. Evolution. 59(1):113–125. 15792232

[CIT0020] Mills S, Alcántara-Rodríguez JA, Ciros-Pérez J, Gómez A, Hagiwara A, Galindo KH, Jersabek CD, Malekzadeh-Viayeh R, Leasi F, Lee J-S, et al. 2017. Fifteen species in one: deciphering the *Brachionus plicatilis* species complex (Rotifera, Monogononta) through DNA taxonomy. Hydrobiologia. 796(1):39–58.

[CIT0021] Moreno E, Conde‐Porcuna JM, Gomez A. 2017. Barcoding rotifer biodiversity in Mediterranean ponds using diapausing egg banks. Ecol Evol. 7(13):4855–4867. 28690814 10.1002/ece3.2986 PMC5496561

[CIT0022] Proios K, Michaloudi E, Papakostas S, Kappas I, Vasileiadou K, Abatzopoulos TJ. 2014. Updating the description and taxonomic status of *Brachionus sessilis* Varga, 1951 (Rotifera: Brachionidae) based on detailed morphological analysis and molecular data. Zootaxa. 3873(4):345–370. 25544227 10.11646/zootaxa.3873.4.2

[CIT0023] Putz H, Brandenburg K. 2006. DIAMOND-crystal and molecular structure visualization. Crystal Impact-GbR, Kreuzherrenstr. 102:53227.

[CIT0024] Radix P, Severin G, Schramm KW, Kettrup A. 2002. Reproduction disturbances of *Brachionus calyciflorus* (rotifer) for the screening of environmental endocrine disrupters. Chemosphere. 47(10):1097–1101. 12137043 10.1016/s0045-6535(01)00335-6

[CIT0025] Ratnasingham S, Hebert PDN. 2007. Bold: the barcode of life data system (http://www.barcodinglife.org). Mol Ecol Notes. 7(3):355–364. 18784790 10.1111/j.1471-8286.2007.01678.x PMC1890991

[CIT0026] Rico-Martínez R, Snell TW, Shearer TL. 2013. Synergistic toxicity of Macondo crude oil and dispersant Corexit 9500A(®) to the *Brachionus plicatilis* species complex (Rotifera). Environ Pollut. 173:5–10. 23195520 10.1016/j.envpol.2012.09.024

[CIT0027] Rublee PA. 1998. Rotifers in arctic North America with particular reference to their role in microplankton community structure and response to ecosystem perturbations in Alaskan Arctic LTER lakes. Hydrobiologia. 387/387:153–160.

[CIT0028] Segers H. 2007. Annotated checklist of the rotifers (Phylum Rotifera), with notes on nomenclature, taxonomy and distribution. Zootaxa. 1564(1):1–104.

[CIT0029] Sladecek V. 1983. Rotifers as indicators of water quality. Hydrobiologia. 100(1):169–201.

[CIT0030] Sørensen MV, Giribet G. 2006. A modern approach to rotiferan phylogeny: combining morphological and molecular data. Mol Phylogenet Evol. 40(2):585–608. 16690327 10.1016/j.ympev.2006.04.001

[CIT0031] Wares JP, Cunningham CW. 2001. Phylogeography and historical ecology of the North Atlantic intertidal. Evolution. 55(12):2455–2469. 11831661 10.1111/j.0014-3820.2001.tb00760.x

[CIT0032] Wilts EF, D. Bruns D, Fontaneto D, Ahlrichs WH. 2012. Phylogenetic study on *Proales daphnicola* Thompson, 1892 (Proalidae) and its relocation to Epiphanes (Rotifera: Epiphanidae). Zool Anz. 251(3):180–196.

